# A scoping review protocol of age-friendly practices during the COVID-19 pandemic

**DOI:** 10.12688/hrbopenres.13619.1

**Published:** 2022-09-30

**Authors:** Viveka Guzman, Paul O'Dwyer, Frank Doyle, Maria Pertl, Ronan Foley, Patricia Morsch, Delfina Alvarez, Enrique Vega

**Affiliations:** 1 School of Population Health, Royal College of Surgeons in Ireland, Dublin, D02DH60, Ireland; 2 Healthy Life Course Unit, Pan American Health Organization, Washington DC, 20037, USA; 3 Department of Geography, National University of Ireland, Maynooth, Maynooth, W23F2H6, Ireland

**Keywords:** Aged, Age-friendly, Environmental intervention, COVID-19, Scoping review, Study protocol

## Abstract

**Background: **Actions focused on age-friendly environments contribute to promote and maintain older people’s functional ability and may enable them to contribute to their communities and enjoy life. As such, age-friendly practices require collaboration between diverse stakeholders across multiple sectors responsible for natural, built, and social environments, which can be particularly relevant during public health emergencies when socio-ecological vulnerabilities become more salient and may disproportionally affect older people. This paper presents a protocol for a scoping review aiming to investigate the breadth of evidence concerning the development, implementation, and evaluation of age-friendly practices during the COVID-19 pandemic. The protocol sets out the objectives, methods, and dissemination plans for the review.

**Methods:** The scoping review will be conducted in line with the Joanna Briggs Institute (JBI) scoping review methodology. We will search databases (PubMed, Web of Science, Embase, CINAHL, Scopus, PsychNet) and grey literature sources. Publications relating to practices across the 8 domains of the World Health Organization’s age-friendly cities and communities’ framework will be included. A tabular data extraction tool will be used to facilitate a narrative synthesis of results.

**Ethics and dissemination:** Ethical approval is not required as the methods proposed for this scoping review consist of collecting publicly available data. Findings will be reported in line with the Preferred Reporting Items for Systematic Reviews and Meta-analyses extension for Scoping Reviews (PRISMA-ScR) and submitted to a journal for academic dissemination. Lay dissemination plans include an infographic and a blog-style article presenting our core results.

**Conclusion: **The publication of this protocol allows for transparency in the systematic process of a scoping review focused on age-friendly practices during COVID-19. Findings emerging from the scoping review will provide insights into the evidence available regarding age-friendly activities during COVID-19 and may inform future age-friendly practices during public health emergencies and beyond.

## Introduction

The COVID-19 pandemic has exposed the fragility and resilience of the services and resources that support older people’s health and well-being such as health-care, social protection, long-term care, information and financial systems ^1^. Older people are considered at high risk from COVID-19 as chronological age has been shown to be associated with increased risk of developing severe illness if infected, which has led to the highest mortality rate among this age group ^2, 3^. Moreover, the public health measures necessary to contain the spread of the virus, including shelter-in-place recommendations, physical distancing, and closure of third spaces, such as community halls, places of worship, libraries and outdoor public places, have significantly influenced the routines of many older individuals globally ^4– 6^. For some older people, these disruptions have been met with periods of adaptation and successful implementation of coping strategies ^7, 8^. However, those facing a high degree of disruption or lacking adequate support may experience long-term consequences in their physical, mental and/or social well-being ^4^. Although individual characteristics have been shown to be significant predictors for these outcomes, emerging evidence indicates that socio-ecological factors embedded at multiple levels of influence play a key role in driving experiences of health and well-being during the COVID-19 pandemic ^9– 16^.

Previously established theoretical perspectives rooted in the field of environmental gerontology recognize ageing as a widely heterogeneous experience and highlight that the benefits of specific environments relate to how well they suit the shifting needs and capabilities of a certain individual through the life-course ^17, 18^. In the context of COVID-19, the physical, social, and economic environments where people were nested before and throughout the public health crisis have shaped individual’s everyday life and determined what they can be and do, given their intrinsic capacities both during and beyond the pandemic. The intrinsic capacity is all the physical and mental capabilities of an individual, divided in five main domains: vitality, sensorial (i.e., visual and hearing), cognitive, locomotor and psychological capacities ^19, 20^. The intersection between an individual’s intrinsic capacity, the characteristics of their environment and how individuals interact with it determine their functional ability ^21^. Functional ability is defined by the opportunities for an individual to be and do what they deem valuable- that is the ability to meet their basic needs, maintain independence, complete every day valued tasks, build and maintain relationships with others, and contribute to society ^21^. According to the World Health Organization (WHO), intrinsic capacity, environments, and functional ability constitute the 3 components necessary to promote and maintain healthy ageing ^22^.

Significantly, public health crises such as COVID-19 can block access to valuable health enabling resources leading to an accelerated decline towards poor health and exacerbation of inequalities in healthy ageing, particularly among vulnerable sub-groups of older people ^4^. The inequalities highlighted by the pandemic and challenges presented raise fundamental questions about the responses needed to support older people during public health crises ^23^. Given the potential of prominent environments such as home, neighbourhood and local community to exert an influence on healthy aging and the delivery of services for older people in these circumstances, a multi-disciplinary approach is needed for the development and implementation of appropriate policies and interventions, considered as age-friendly practices. The development of such age-friendly practices requires actions in multiple sectors, and calls for multi-stakeholder engagement that includes older people, multiple levels of government, service providers, civil society, community groups, and friends and families ^24, 36^. 

Responding to the need for guidance to develop appropriate approaches, in 2007 the WHO published a guide for cities to develop age-friendly cities and communities based on a framework of 8 interconnected domains: (1) outdoor spaces and infrastructure; (2) transportation; (3) housing; (4) social participation; (5) respect and social inclusion; (6) civic participation and employment; (7) communication and information; and, (8) community support and health services ^25^. Following the publication of the age-friendly cities guide, in 2010 the WHO launched the Global Network for Age-Friendly Cities and Communities with the objective of building a platform of shared experiences around the globe and catalysing progress through political commitment, planning, action and evaluation of age-friendly practices ^24^. Later in 2015, the WHO’s Age-Friendly framework was expanded to emphasize a broader notion of age-friendly environments encompassing urban, rural, and suburban communities ^24^.

The development of appropriate responses to rapidly changing environments due to COVID-19 has required adaptation of existing support strategies and leveraging of existing infrastructure in cities and communities ^19^. Evidence of feasible and accessible age-friendly practices during the pandemic is emerging from the Global Network of Age-Friendly Cities and Communities ^26– 28^. Such evidence has the potential to increase the quality of planning of concrete age-friendly actions during current and future public health emergencies ^26^. However, to our knowledge a comprehensive review of age-friendly practices during the COVID-19 pandemic has not been carried out to date, and synergies with other public health and environmental interventions remain unexplored. Thus, the objective of our scoping review will be to investigate the development, implementation, and evaluation of age-friendly practices during COVID-19. The aim of this paper is to present a protocol for the proposed scoping review by detailing our objectives, methods, and plans for reporting results. Publication of this protocol allows for transparency of the review process, and it also reduces the potential for duplication, facilitates peer review of the planned methodology before it is carried out, and offers the researchers the opportunity to plan the resources and logistics necessary to complete the review ^29, 30^. 

## Methods

Methods will be based on the Joanna Briggs Institute (JBI) guidance for conducting a scoping review ^31^. A scoping review is defined as “a form of knowledge synthesis that addresses an exploratory research question aimed at mapping key concepts, types of evidence, and gaps in research related to a defined area or field by systematically searching, selecting, and synthesizing existing knowledge” ^32^. A scoping review was chosen to ascertain the diverse approaches that may be represented across the multi-dimensional domains that comprise age-friendly environments within the existing international literature. This approach will allow us to characterize the interventions and support strategies developed across the 8 domains of the age-friendly framework, which may differ widely in delivery methods, resources needed and adaptative strategies deployed during the pandemic. An additional benefit of our scoping review is that we will consider practices and interventions that may provide innovative age-friendly approaches but that are conducted outside the global network of age-friendly cities and communities. This approach may identify potential synergies with other public health and environmental interventions that promote the development of health enabling environments, such as healthy cities ^33^, future- and age-ready cities ^34, 35^, and sustainable cities ^36^.

A comprehensive search strategy will be utilised to identify experiences on the development, adaptation and implementation of age-friendly support strategies and interventions during the COVID-19 pandemic from electronic databases, grey literature sources, and reference list screening of publications dating from January 2020 to date. The research questions that our scoping review seeks to address are:

- What is the nature of the evidence relevant to the development of age-friendly practices for older people in the COVID-19 context?
- What are the characteristics of practices across the 8 age-friendly domains that have been adapted or developed during the COVID-19 pandemic? For instance:
  ○ Which age-friendly domains have been targeted to a higher degree and which have received less attention?
  ○ In what geographical contexts have age-friendly practices been implemented?
  ○ What rationale and outcomes are considered for age-friendly practice development and/or evaluation during COVID-19?
  ○ What are the profiles of older people’s needs that these age-friendly practices seek to address?
  ○ What factors facilitate or hinder the implementation of age-friendly practices in the pandemic context?

### Identification of relevant evidence

Relevant publications in English, Spanish, French or Portuguese will be selected according to key inclusion criteria identified through the Population, Concept, Context (PCC) screening criteria.

*Population:* The population of interest is comprised by individuals chronologically aged 60 years old and over, or who are otherwise identified as older people ^37^.

*Concept:* Age-friendly practices typically aim to foster one or more functional abilities, and may include policies, systems, products and services that foster healthy ageing ^24^. As such, age-friendly practices may be defined as actions that promote health and physical and mental well-being across the life course, as well as actions that aim to remove physical and social barriers for older people to continue to take part in valued functioning, even if they experience intrinsic capacity loss ^38^. Our scoping review will consider interventions and support strategies across the 8 domains of age-friendly communities stated beforehand ^25^.

*Context:* The context of this review comprises any community/city/region in the world at any stage of the COVID-19 pandemic.

Further details of inclusion and exclusion criteria are shown in Table 1.

**Table 1.** Inclusion and exclusion criteria of evidence.

Inclusion	Exclusion
Report or study detailing interventions or support strategies: - for individuals aged 60 and over - whereby the mean age of participants falls above 65 years of age - individuals otherwise identified as older people	Report or study detailing interventions or support strategies for younger populations or where the mean age is below 65 years old
Intervention or support strategies across any of the 8 domains of age-friendly communities	Intervention or support strategies outside the 8 domains of age-friendly communities (i.e., individual psychotherapy)
Practices referring to ecological, psychosocial, cognitive, or physical measures that promote healthy aging	Practices that do not concern any aspect of healthy aging
Full text peer-reviewed and full text non-peer reviewed publications	Conference abstracts or keynotes where it is not possible to access detailed characteristics of the intervention or support strategy developed
Age-friendly practices and/or practice components developed and/or adapted to face the needs and challenges presented by the COVID-19 pandemic	Pre-COVID-19 age-friendly practices with no adaptations during the pandemic

### Types of sources

The proposed scoping review will consider all study designs utilizing either quantitative and/or qualitative approaches. Additionally, we expect a significant amount of literature beyond academic publications might detail the experiences of local authorities, community organizations, older people, and other stakeholders with age-friendly practices during the pandemic. Thus, our review will also consider grey literature publications such as governing documents, program evaluations, reports, and technical documentation.

### Search strategy

Relevant documents will be identified through four steps: (1) A limited electronic search will be carried out in two online databases (PubMed, Web of Science) and followed by an analysis of relevant words in the title and abstract, as well as index terms used to describe the articles; (2) A comprehensive search using all identified keywords will be carried out in all included databases (Web of Science, PubMed, Scopus, PsychNet, Embase, and CINAHL). The search strategy comprising relevant terms will be optimized for each database and/or information source.; (3) A grey literature search will be carried out comprising the WHO Global Database of Age-Friendly Practices ^38^, Google searches utilizing keywords, and targeted website search of known Age-Friendly Organizations (i.e., the American Association of Retired Persons- AARP); and, (4) The reference list of all evidence sources included will be screened to identify additional reports or studies. Where appropriate, authors of publications will be contacted to request missing or additional data.

The initial search strategy that will be used when searching the PubMed data base is presented in Table 2. This initial search strategy was developed in consultation with a librarian at the Royal College of Surgeons in Ireland. Further iterations of the search strategy will continue to seek the librarian’s advice to ensure key literature is captured.

**Table 2.** Relevant search terms and initial search strategy.

Population, Concept, Context (PCC)	Search terms *MeSH/DeCS
**Population: older** **population**	*Aged*, older people, older individuals, older population, ageing, elderly, senior
**Concept: age-friendly** **practices**	GENERAL: Age-friendly, ageing-in-place, active ageing *, healthy aging, independent living* AGE-FRIENDLY DOMAINS: *transportation, commuting*, mobility, outdoors, *built environment,* *neighbourhood characteristics, social environment,* community support, *health services, communication,* information, civic participation, *employment*, volunteering, *social inclusion, social participation, housing,* *home environment* PRACTICE: Practice, program, intervention, support strategy
**Context: COVID-19**	*COVID-19,* 2019 Novel Coronavirus Disease, 2019 Novel Coronavirus Infection, 2019-nCoV Disease, 2019-nCoV Infection, COVID-19 Pandemic, COVID-19 Pandemics, COVID-19 Virus Disease, COVID-19 Virus Infection, COVID19, Coronavirus Disease 2019, Coronavirus Disease-19, SARS Coronavirus 2 Infection, SARS-CoV-2 Infection, Severe Acute Respiratory Syndrome Coronavirus 2 Infection
**Initial PubMed search** **strategy according** **to PCC**	(COVID-19 OR Coronavirus OR Covid-19) AND (aged OR "older people" OR elder* OR senior OR "older population*") AND ("age-friendly" OR transport* OR hous* OR dwell* OR communication OR information OR "social inclusion" OR "social participation" OR "civic participation" OR "employment" OR "work" OR "income" OR "outdoor*" OR "enabling environments" OR "built environment" OR build*) AND (practic* OR service* OR intervention* OR support* OR strateg* OR program*)

*MeSH: Medical Subject Headings; DeCS: Health Science Descriptors

### Evidence selection

Titles and abstracts will be screened for inclusion, and full text publications of those that appear to meet the inclusion criteria will be retrieved and screened for inclusion. Since abstracts are often unavailable in grey literature documents ^39^, screening will be conducted on items’ abstracts, executive summaries, or table of contents, according to availability. Potentially relevant sources will be imported into the bibliographic reference manager Mendeley, and any duplicates removed. Two reviewers will independently screen the evidence available to ensure consistency. The online tool COVIDENCE will be used at this stage of the review. Any disagreements will be solved by consensus or with help from a third reviewer. Once suitable papers have been selected, full text screening will be carried out by the lead reviewer on all articles that meet the inclusion criteria. Reasons for exclusion of sources of evidence at full text that do not meet the inclusion criteria will be recorded and reported in the scoping review. The results of the search and evidence inclusion process will be reported in full in the final scoping review and presented in a Preferred Reporting Items for Systematic Reviews and Meta-analyses extension for scoping review (PRISMA-ScR) flow diagram ^40^.

### Data extraction

A data charting table will be developed, piloted, and refined from early stages of the review with the objective of recording key information from the source documents. A preliminary overview of the items included in the data extraction tool is provided in Table 3. The charting tool will be revised and updated as necessary during the process of extracting data from each included evidence source. Modifications will be detailed in the scoping review.

**Table 3.** Preliminary items included in the scoping review extraction tool.

**Overview**	*ID*	Practice identification number for the purpose of scoping review
	*Name*	Name of the practice as in original publication
	*Period of implementation*	Date practice started and current status (i.e., ongoing or finished)
**Geographical** **and demographic** **context**	*Global region*	Global region where practice was developed/implemented
	*City/Community*	City or community where practice was developed/implemented
	*Population size (% of older people)*	Total population of city/community (percentage of older people in the city/community)
**Concept of age-** **friendly practice**	*Domain*	Pertaining age-friendly domain(s) according to WHO Age-Friendly framework
	*Target population*	General or sub-groups of target population
	*Practice description*	Overview of the practice
	*Established evaluation process*	Details on the evaluation process (available: no/yes, if yes, detail the planned process and/or results)
**Resources**	*Inputs*	a) Human resources (i.e., practice lead; involvement/collaboration of older people in design/implementation; role of other stakeholders) b) Material resources needed for practice development/implementation
	*Outputs*	Desired and observed outputs
	*Enablers*	Factors that facilitate the development/implementation of the practice
	*Challenges*	Factors that hinder the development/implementation of the practice

### Collating, summarizing, and presenting the results

A narrative synthesis approach will be employed to answer the research questions. Narrative synthesis is an approach where researchers rely primarily on the use of words and text to summarise and explain the findings from an evidence synthesis ^41^. This will be accompanied with a table that synthesises the core information retrieved with the extended data collection tool (see Table 3). Findings from our scoping review will be submitted to an academic journal and conference presentations for dissemination. A lay dissemination plan will also be developed including an infographic and blog style article.

### Study status

At time of publication of this protocol, we are finalizing search term trials and preparing to carry on full database searches. 

## Discussion

This protocol allows for transparency in the process of a scoping review focused on age-friendly practices during COVID-19, as it pre-defines our objectives, methods, and plans for reporting of results ^29^. Additionally, we have detailed our inclusion and exclusion criteria, approach to identify relevant data and how the data will be extracted. In line with JBI guidance, any deviations of the scoping review from the protocol will be clearly highlighted and explained in the publication of results from the scoping review ^29^. The publication of the protocol is important in limiting the occurrence of reporting bias and may also be a useful tool for other researchers preparing projects of evidence-synthesis related to healthy ageing and/or age-friendly practices. As data, research and innovation are among the enablers of the Decade of Healthy Ageing ^42^, providing detailed information on the methodologies to be used may also support the development of further research by identifying research priorities and gaps in evidence. Moreover, our project responds to the Decade of Healthy Ageing call for synthesis of evidence that has potential to benefit older people, their families, and communities and that also facilitates sharing of experiences and ways to scale up good practices to reach more people ^42^.

Evidence emerging from the scoping review proposed in this protocol has the potential to support age-friendly cities and communities by summarizing the experiences of development and implementation of practices across multiple age-friendly domains in the context of the COVID-19 pandemic. This information may facilitate the total or partial replication of age-friendly practices in new contexts. Additionally, it may underscore gaps and opportunities for policies, environmental interventions and public health programmes needed to support older people. Lessons drawn from the development and implementation of age-friendly practices during COVID-19 also provide useful insights for multisectoral collaborations and the development of future emergency preparedness plans.

For instance, the adaptation of age-friendly initiatives established previous to the pandemic may facilitate a fast mobilization of community networks that work alongside older people to maintain the functioning that are most valuable to them even during the context of the COVID-19 pandemic ^26^. Significant scope also exists for the development of new innovative strategies that are low-cost and may be implemented in multiple settings including those characterized by low availability of human and/or financial resources. It is also relevant to identify practices that do not self-identify as age-friendly but may share a vision of promoting health enabling environments for all through the life-course. This will allow to identify potential synergies between existing programs and build further collaboration towards resilient systems that leave no-one behind. It is likely that age-friendly practices will become increasingly significant in an ageing world that is facing the opportunities and challenges brought-up by parallel trends of increasing urbanization, environmental degradation and rise of infectious disease outbreaks.

## Conclusion

This protocol presents our plans for a scoping review that will contribute to the evidence base of age-friendly practices developed during the COVID-19 pandemic. The protocol sets up the context and methods within which the review will be carried out and facilitates a systematic and transparent process that will minimize risk of bias and allow the research team to define next steps accordingly. Moreover, the publication of the scoping review protocol may provide further guidance to researchers interested in developing similar approaches that follow a systematic evidence-based strategy. We consider findings emerging from the scoping review proposed will inform the development of age-friendly practices across the globe and guide future research that seeks to address knowledge gaps concerning interventions for older people in emergency situations and beyond.

## Ethics

Ethical approval will not be needed as the scoping review outlined within this protocol will use publicly available literature.

## Data Availability

No data are associated with this article.
