# Supplementary material for: A scoping review protocol of age-friendly practices during the COVID-19 pandemic
Source: HRB Open Res. 2022 Sep 30;5:64. [Version 1] doi: 10.12688/hrbopenres.13619.1 (PMC10170180; doi:10.12688/hrbopenres.13619.1)
Supplement: Peer review history

# Peer review: Reviewer response for version 1

**DOI:** 10.21956/hrbopenres.14891.r33486

**Published:** 2023-05-09

**Authors:** Katie Robinson

**Affiliations:** 1 School of Allied Health, Faculty of Education and Health Sciences, Health Research Institute, Ageing Research Centre, University of Limerick, Limerick, County Limerick, Ireland

Congratulations on developing this protocol for a robust scoping review on an important topic. 

 The introduction presents a strong argument and rationale for the proposed review. Some further reference to the unique challenges older adults faced compared to other age groups during the pandemic and the role of ageism in shaping policy responses to this age group would further enhance this introduction. 

 The methods of the scoping review are very well presented in general. Given the review is focused on age friendly practices during the COVID-19 pandemic, I think the team could consider data extraction on the stage of the pandemic and public health restrictions during those stages. This contextual information may be important as practices developed before widespread availability of vaccinations or when shelter-in-place restrictions were in place may vary widely from practices in more recent months. 

 Stating explicitly that the search strategy will be refined between step 1 and 2 might be useful. 

 The search strategy presented in table 2 was developed in conjunction with a librarian which is positive however it would be useful to know what literature informed the development of this strategy. 

 The definition of  your concept (age-friendly practices) is very broad and could require the use of a very large number of search terms and could lead to the inclusion of very large number of studies/ reports in the review. Could this definition be refined a little further?

Is the study design appropriate for the research question?

Yes

Is the rationale for, and objectives of, the study clearly described?

Yes

Are sufficient details of the methods provided to allow replication by others?

Partly

Are the datasets clearly presented in a useable and accessible format?

Not applicable

Reviewer Expertise:

I have experience of conducting scoping reviews and am familiar with the literature on older adults experiences of the pandemic.

I confirm that I have read this submission and believe that I have an appropriate level of expertise to confirm that it is of an acceptable scientific standard.

# Peer review: Reviewer response for version 1

**DOI:** 10.21956/hrbopenres.14891.r33247

**Published:** 2023-01-19

**Authors:** Ione Avila-Palencia

**Affiliations:** 1 Centre for Public Health, Queen's University Belfast, Belfast, UK

PREVIEW:

 A scoping review protocol of age-friendly practices during the COVID-19 pandemic.

 SUMMARY:

 The aim of this paper is to present a protocol for a scoping review about investigating the development, implementation, and evaluation of age-friendly practices during COVID-19.

 The manuscript is easy to read and presents a well-explained framework in which to rely on.

 GENERAL COMMENTS:

 The rationale and the objectives are clearly described and all the different steps of the review are clearly stated. To be honest, I don’t have much to add. I think the protocol is pretty clear and it will be very useful to have it indexed by when the review is carried on.

Is the study design appropriate for the research question?

Yes

Is the rationale for, and objectives of, the study clearly described?

Yes

Are sufficient details of the methods provided to allow replication by others?

Yes

Are the datasets clearly presented in a useable and accessible format?

Not applicable

Reviewer Expertise:

Environmental epidemiology, urban health

I confirm that I have read this submission and believe that I have an appropriate level of expertise to confirm that it is of an acceptable scientific standard.
